# Assessing Feasibility of an Early Childhood Intervention Using Mobile Phones Among Low-Income Mothers of Newborns: Qualitative Interview Study

**DOI:** 10.2196/17179

**Published:** 2020-05-28

**Authors:** Donglan Zhang, Lan Jin, Di Liang, Ruijin Geng, Yun Liu, Yu Ling, Fan Jiang, Yunting Zhang

**Affiliations:** 1 Department of Health Policy and Management College of Public Health University of Georgia Athens, GA United States; 2 Center for Intercultural Learning, Mentorship, Assessment and Research Purdue University West Lafayette, IN United States; 3 School of Public Health Key Lab of Health Technology Assessment, National Health Commission Fudan University Shanghai China; 4 Clinical Research Center Morehouse School of Medicine Atlanta, GA United States; 5 Kunming Children’s Hospital Kunming China; 6 Child Health Advocacy Institute, National Children’s Medical Center Shanghai Children’s Medical Center Shanghai China; 7 MOE-Shanghai Key Laboratory of Children's Environmental Health Xin Hua Hospital Affiliated to Shanghai Jiao Tong University School of Medicine Shanghai China

**Keywords:** mobile health, interview, health belief model, early child development

## Abstract

**Background:**

Many children aged younger than 5 years living in low- and middle-income countries are at risk for poor development. Early child development (ECD) programs are cost-effective strategies to reduce poverty, crime, school dropouts, and socioeconomic inequality. With the spread of low-cost mobile phones and internet access in low- and middle-income countries, new service delivery models such as mobile phone–aided interventions have a great potential to improve early childhood development.

**Objective:**

This study aimed to identify the beliefs on importance of ECD, feasibility of a proposed intervention using mobile phones and factors that may affect the usability of the intervention among mothers of newborns in a poverty-stricken area in southwestern China.

**Methods:**

We conducted an in-depth, semistructured interview study of 25 low-income mothers of newborns recruited from two county hospitals in Yunnan Province. We applied the health belief model and cultural competence theories to identify the facilitators, barriers, and preferences among the target population for parenting knowledge.

**Results:**

The results showed that the participants had low health literacy and high perceived needs for learning ECD knowledge. At the same time, they experienced several barriers to learning parenting information and following evidence-based instructions including having limited time, limited financial resources, and different opinions on childcare among family members. Many participants preferred to receive personalized messages tailored to their specific needs and preferred videos or graphics to text only in the messages. Many favored a separate module to support postpartum mental health.

**Conclusions:**

The study assessed the acceptability of an early childhood intervention using mobile phones to meet the needs of the target population based on their beliefs, traits, and preferences and provided suggestions to refine the intervention to improve its usability.

## Introduction

Early child development (ECD) has been shown to link with long-term health outcomes and well-being. Between 2004 and 2010, the number of children aged younger than 5 years who were at risk for poor development in low- and middle-income countries (LMICs) decreased from 279.1 million to 249.4 million [1-4]. There were 17.43 million at-risk children in China, and geographic disparities remained significant [1]. Prevalence of developmental delays in a low-income rural county in Northeast China was found to increase from 13.4% when the infants reached 6 to 12 months to 50.4% when they reached 24 to 30 months, while in most urban areas of China this prevalence remained under 10% [5,6]. In certain regions of China, risk factors for developmental delays such as inadequate learning resources and activities were prevalent [7]. A household survey conducted among a representative sample of children aged 3 to 6 years in southwestern China found that 72% of the primary caregivers had not played with their children and 47% did not read to them in the past year [8].

ECD programs are cost-effective strategies to reduce poverty, crime, school dropouts, and socioeconomic inequality [9-12]. Targeted interventions have been implemented in the poorest areas of China focusing on nutrition supplement and early stimulation [13]. Parenting education provided in health check-ups, home visiting, and ECD centers have shown to be efficacious in some pilot studies [7]. However, it is challenging to scale up those programs due to limited resources available for a population of huge density, especially in remote areas where medical professionals are in short supply.

Currently, with the spread of low-cost mobile phones and internet access in LMICs, new service delivery models such as mobile phone–aided interventions (mHealth) have a great potential to influence ECD at the population level [14,15]. Short message services (text messages) and smartphone apps have proven to be successful in changing parents’ beliefs and behaviors surrounding early child development. The intervention for parenting education covers a wide range of topics such as breastfeeding, nutrition, developmental milestones, anticipatory guidance, language and cognition-oriented activities, car seats, fire safety, etc. However, it has been found that compared with well-off families, caregivers with lower income, education, and literacy were less likely to enroll in mHealth programs, so the programs were of less benefit to the low-income people [16].

Previous studies identified possible incentives for low-income people to engage in mHealth programs, including financial incentives and need for better health for their children. However, barriers were also found such as culture and beliefs, limited battery life of mobile phones combined with the lack of readily available electricity, rates of interrupted mobile phone services, mistrust or stronger desire to preserve privacy, and lower technological literacy [16-19]. These issues must be elucidated and addressed before well-designed interventions can reach their intended audience and produce meaningful public health outcomes. Previous reviews have found that most effective public health programs are based on an understanding of the health behaviors and the contexts in which they occur with behavior change theories [20]. The health belief model (HBM), one of the most widely used theoretical models of health behaviors, has been applied most often for health concerns that are prevention-related and asymptomatic, where beliefs are as important or more important than overt symptoms [21]. As early child development lies more on the desire of parents of achieving better health and development rather than just management of certain diseases, we believe it is most suitable for our study.

This study aimed to identify the demand for ECD knowledge, acceptability of a promising ECD intervention using mobile phones, and factors that may affect the usability of the intervention among mothers of newborns in a poverty-stricken region of China. We employed a qualitative research method in this study because the rich data obtained through this method regarding participants’ perceptions, preferences, and barriers are highly aligned with the study’s purpose and scope. Results from this study will be used to support the design and implementation of the ECD intervention using mobile phones.

## Methods

### Interview Sites

In March 2019, we conducted 25 semistructured in-depth interviews among mothers of newborns recruited from two county hospitals in Yunnan Province, southwestern China: the Maternal and Child Health Hospital in Nanjian County and the People’s Hospital in Jinggu County, both large hospitals where the majority of infants in the two counties were delivered. These two counties compose China’s most poverty-stricken area based on the percentage of people below the national poverty line. The study was approved by the institutional review board at the University of Georgia (STUDY00005670).

### Theoretical Framework

We applied the HBM and cultural competence theories to identify the preferences and barriers among the target population for receiving and practicing evidence-based parenting knowledge [22]. HBM has been used in a broad range of behavioral interventions including interventions to increase screening, reduce risky behavior, and improve adherence to medication regimen [23-26]. In its latest version, the key constructs of the model include cues to action and self-efficacy and perceived susceptibility, severity, benefits, and barriers [27]. Studies have shown that parental perception regarding the healthy development of their young children often involves perceived barriers to take action [28-30]. Behavioral change will not occur if parents’ perceived barriers outweigh perceived benefits [31].

**Figure 1:**
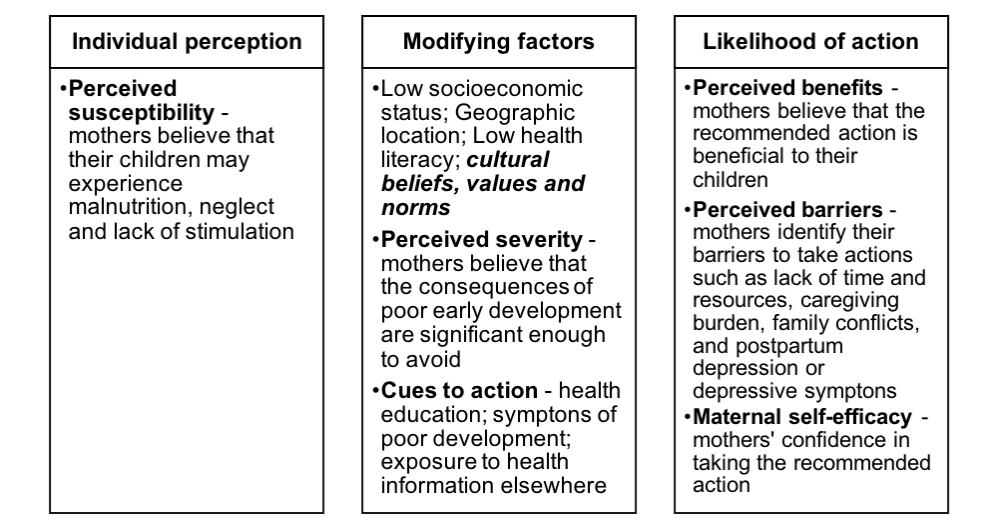
Theoretical framework based on the health belief model.

In addition, although our target population did not have language barriers when reading ECD messages, they might have cultural beliefs that were not consistent with the evidence that supports child healthy development. Culturally competent approaches recognize and respect the cultural beliefs, norms, and values of the target population and aim to improve effectiveness of the intervention [32,33]. Therefore, in our theoretical framework, cultural beliefs and norms were included as a modifying factor (Figure 1).

### Study Sites and Participants

The inclusion criteria were the study participants should be in good health with no severe delivery complications or clinical diagnosis of postpartum depression and have a healthy infant aged less than 1 month.

Two physicians assisted in identifying and recruiting potentially eligible participants through the hospital wards. The 25 participants were recruited in the local hospitals through criterion sampling. We reached sample saturation with 25 participants, until a point where additional interviews did not develop new theoretical insights. In other words, sampling was stopped when the data were repetitive and our research questions had been answered. To examine their eligibility for the study based on the criteria, electronic medical records were retrieved to scan for any recorded delivery complications or diagnosed depression. Informed consents were obtained from all participants who met the inclusion criteria and agreed to participate in the study.

### Data Collection

Two trained interviewers (DZ and YZ) conducted the in-depth interviews in private rooms at the hospitals. We developed an interview guide based on the theoretical framework (Figure 1) pertaining to the design of the mobile health intervention. These topics included (1) mothers’ individual perceptions about the importance of avoiding adversities in the early years; (2) benefits and barriers when feeding and nurturing newborns according to recommendations sent over the phone; (3) family socioeconomic status, family structure, relationship, beliefs, and values related with adhering to the recommendations; (4) concerns about privacy; and (5) preferences for the frequency of receiving messages. During the interview, the Edinburgh Postnatal Depression Scale, Chinese version, was used to evaluate mothers’ mental health status [34,35]. Participants were shown sample text messages and were asked questions about the messages. The message included about 600 Chinese characters and two pictures with four sections: (1) weekly achievement, showing easily identifiable developmental milestones; (2) weekly care, providing relevant information on baby’s daily care; (3) weekly game, showing examples of age-paced games and parent-child activities including playing games, singing nursery rhymes, reading, and drawing; and (4) weekly question and answer, providing answers to common questions asked by parents. The interview was designed to begin with sociodemographic questions followed with questions according to each construct in the HBM.

Interviews were conducted on a one-on-one basis. Each interview lasts approximately 60 to 90 minutes. To aid in recruitment, ¥100 (US $15 in 2019) was provided to each participant. All interviews were tape recorded with participant permission.

### Data Analysis

Interviews were transcribed verbatim and entered into qualitative analysis software NVivo 12 (QSR International). A coding structure based on the interview guide was iteratively developed to label specific content from the interviews. The transcripts were coded separately by two members of the study team (DL and RG). After coding, the two team members met to discuss the results and resolve any disagreements in coding by consensus.

## Results

### Participant Demographic Characteristics

Table 1 shows the characteristics of the 25 participants. Their average age was 28 years. More participants lived in urban regions than in rural regions (56% [14/25] vs 44% [11/25]). Most participants (10/25, 40%) were ethnically Han Chinese, 32% (8/25) were Yi, and 16% (4/25) were Dai Chinese. Overall, 48% (12/25) of newborns were male. The majority of the infants’ primary caregivers (18/25, 72%) were mothers, while 12% (3/25) were grandparents. A total of 40% (10/25) of the mothers finished middle school, and 28% (7/25) obtained some college education, while 16% (4/25) finished only elementary school. Half of the participants (13/25, 52%) were unemployed, and 28% (7/25) had a full-time job; 48% (12/25) of participants reported that the main source of family income was from their husband. About half (13/25, 52%) of the participants had household income below ¥5000 (US $750) per month, whereas 52% (13/25) of them reported they were willing to spend between ¥1000 (US $150) and ¥2000 (US $300) on childcare, and 28% (7/25) reported they were willing to spend more than ¥2000 (US $300). A majority of the participants (23/25, 92%) were not receiving public financial aid. Most (23/25, 92%) were married, and 40% (10/25) lived in extended families with 7 or more family members.

**Table 1:** Participant demographic characteristics (n=25).

Characteristics		Value
Age in years, mean (SD)		28.08 (6.55)
**Region, n (%)**		
	Urban or township	14 (56)
	Rural	11 (44)
**Race/ethnicity, n (%)**		
	Han	10 (40)
	Yi	8 (32)
	Hui	2 (8)
	Dai	4 (16)
	Yao	1 (4)
**Sex of the newborn, n (%)**		
	Male	12 (48)
	Female	11 (44)
	No response	2 (8)
**Primary caregiver of the baby, n (%)**		
	Self (mother of the baby)	18 (72)
	Grandparents of the baby	3 (12)
	Self and grandparents	3 (12)
	No response	1 (4)
**Education of the mother, n (%)**		
	Elementary school	4 (16)
	Middle school	10 (40)
	High school	1 (4)
	Some college	7 (28)
	Four-year college	3 (12)
**Employment of the mother, n (%)**		
	Full-time employment	7 (28)
	Self-employed	5 (20)
	No employment	13 (52)
**Household main income source, n (%)**		
	Double income	7 (28)
	Husband’s income	12 (48)
	Family farm or business	4 (16)
	Income from parents-in-law	1 (4)
	No response	1 (4)
**Estimated household income per month (** **¥** **), n (%)**		
	<5000	13 (52)
	5000-10,000	8 (32)
	≥10,000	4 (16)
**Expenditures on the baby per month (** **¥** **), n (%)**		
	<1000	2 (8)
	1000-2000	13 (52)
	≥2000	7 (28)
	Don’t know	3 (12)
**Receiving government financial aid, n (%)**		
	Yes	2 (8)
	No	23 (92)
**Marital status, n (%)**		
	Married	23 (92)
	Married but live separately	1 (4)
	Married with no official certificate	1 (4)
**Number of family members in the household, n (%)**		
	3 or 4	5 (20)
	5 or 6	10 (40)
	7 and more	10 (40)
**Number of children in the household, n (%)**		
	1	5 (20)
	2	18 (72)
	3	2 (8)

### Participant Health, Work, and Breastfeeding Behavior

Table 2 shows participant health status and lifestyle. All participants self-reported having good health. Most participants had a low risk of postpartum depression with a depression scale score below 13, but 6 people (24%) had a relatively high risk of depression, although no clinical diagnosis of depression was reported. A total of 76% (19/25) of participants reported light to moderate workload for doing housework, and 64% (16/25) of participants would spend more time on childcare than on other things, while 20% (5/25) reported that they would spend more time on family business, housework, or farm work. Most participants (16/25, 64%) had no difficulty in breastfeeding, while 28% (7/25) did not start breastfeeding, and one participant reported having no breastfeeding plan because she had hepatitis B. A majority of the participants (18/25, 72%) planned to breastfeed for 6 months to 1 year, and 12% (3/25) planned to breastfeed for less than 6 months.

**Table 2:** Mothers’ health, work, and breastfeeding behavior (n=25).

Characteristic		Value, n (%)
Self-reported health status, good health		25 (100)
**Depression scale**		
	Yes (≥13)	6 (24)
	No (<13)	19 (76)
**Housework load**		
	Light	19 (76)
	Heavy	4 (16)
	Don’t do housework	1 (4)
	No response	1 (4)
**Time allocation on childcare and other things**		
	Spend more time on business, housework, or farm work	5 (20)
	Spend more time on childcare	16 (64)
	Spend equal amount of time on childcare and work	3 (12)
	No response	1 (4)
**Breastfeeding status**		
	Having no difficulty in breastfeeding	16 (64)
	Not having started breastfed	7 (28)
	Formula feeding	1 (4)
	No plan to breastfeed	1 (4)
**Breastfeeding plan**		
	6 months	3 (12)
	6 months to 1 year	18 (72)
	More than 1 year	2 (8)
	No plan to breastfeed	2 (8)

### Interview Themes

#### Perceived Needs for Learning Early Child Development Knowledge

Around half of participants were concerned about baby’s health issues such as having a cold, fever, acute diseases, adverse reaction to vaccination, tobacco exposure, sleep, and other illnesses, and 3 participants worried about baby’s behavioral problems—for instance, they expressed concerns about their child being spoiled by grandparents and developing bad habits (Multimedia Appendix 1). Two participants expressed the need to learn baby’s cognitive development, and another 2 participants worried about safety issues. Most participants thought it was necessary to learn ECD knowledge. When asked to describe interesting topics in ECD, most participants mentioned baby’s growth and milestones, nutrition, breastfeeding, and complementary feeding. Some participants mentioned that they were interested in parent-child interactions and baby’s social-emotional development. Several participants were interested in mental health support for mothers. Sample responses included: “I want to learn whether his growth is normal, and how parents should interact with him.” “My first child often caught cold probably because I did not have enough breastmilk. For my second child I want to know how to prevent cold” and “I would like to learn about safety issues. My home is near the street. It is dangerous for the kids. I worry about safety when he starts to learn walking.”

Some participants reported that they did not learn parenting knowledge before giving birth to this child, whereas many learned parenting knowledge from books and the internet, and only 2 reported having learned ECD knowledge from health care professionals. A majority of the participants perceived that they had limited ECD knowledge, and some didn’t feel the need to learn parenting knowledge. One response was, “I want to know more about how to take care of my baby when he is getting old, and how to help him recover without taking medicines when he is sick.” Some participants thought that parenting information they found online was inconsistent, and they perceived the necessity to promote the child’s early development.

#### Perceived Usefulness and Barriers to Learn and Practice Parenting Knowledge

Many participants were willing to learn ECD knowledge from health professionals. One response was, “I want to learn parenting knowledge from experts. If it doesn’t take a lot of time, I am willing to follow experts’ suggestions. I can read the messages after my baby sleeps at night. I don’t have time during the daytime.”

Some participants responded that they did not have barriers in following experts’ advice, but many expressed that they had difficulties following experts’ suggestions because they had limited time and financial resources, limited experiences, and their children did not react as expected or they had different opinions with family members. Some examples were, “I have to go to work. I may not have time to follow experts’ suggestions on childcare,” “I think the biggest obstacle for me is that if the experts recommend a new-brand formula which is very expensive, I will not follow their suggestions,” and “I may not follow the experts’ recommendation when my child’s grandparents have different opinions on parenting.”

#### Maternal Self-Efficacy

Many participants illustrated that they made decisions on childcare by themselves or together with their husband. Some participants responded that their husband made the decision about childcare, and some illustrated that their mother-in-law made the decision.

Overall, most participants said they had good relationships with family members, while some expressed that they did not have good relationships with their parents-in-law. Many participants said they preferred to follow health professionals’ suggestions in caring for their babies when there was a conflict between experts’ and family members’ suggestions, while 2 participants replied that they preferred to follow family members’ suggestions. However, only 4 participants were willing to communicate with their family members the knowledge they learned from professionals or persuade them to take the evidence-based advice, and some participants said they would not persuade family members because their parents had limited ability to understand the knowledge.

#### Preferences for the Design of the Intervention

Almost all participants had a smartphone and had access to internet on their phones, and only 1 person shared the smartphone with her husband. Almost all participants preferred to receive messages via smartphone apps, and half of them preferred to receive the messages once a week, while 9 participants preferred to receive the messages every day or twice a week. Overall, most participants responded that they would keep and share the messages and won’t delete them. A majority of the participants were willing to receive personalized information based on their child’s characteristics. One response was, “I would prefer to receive personalized suggestions. My child is different from other kids.” However, several mothers preferred to receive general messages and stated that they would like to keep their baby’s information confidential.

When asked if they were willing to search for answers from health professionals when having questions, many participants preferred to consult with pediatricians at the county hospital, some preferred to communicate via phone with professionals in large hospitals remotely, and some of them would search for answers online. Most of them expressed that they did not trust village doctors and were more likely to ask for opinions from doctors at larger health care facilities.

#### Raised Suggestions to Modify the Message Contents

When shown the sample text message, 11 mothers spent on average less than 3 minutes to read one message, and 10 mothers spent 3 to 5 minutes to read one message, and 4 of them spent more than 5 minutes.

Some participants emphasized that they were very interested in the weekly game section. One participant illustrated, “parent-child interaction is very beneficial for the healthy development of the child’s personality.” Another participant described that “I like the section of parent-child interaction. Every mom enjoys playing games with their child.” Some mothers expressed that they liked the weekly care section on nurturing, teething, or mood management.

Most participants considered the message understandable, and some indicated that they preferred videos and pictures to text. A few said that they did not understand even though they were literate enough to read the text. One response was, “it may be better if the messages can be conveyed via a video. I can easily understand the messages by watching a video.” Further, 3 participants suggested that the messages should include a platform for interaction between receivers and health care professionals. One response was, “I like the weekly question and answer section because I would like to have a platform where specific questions are answered by the experts.”

## Discussion

### Principal Findings

This study focused on assessing feasibility of an ECD intervention using mobile phones to meet the needs of mothers of newborns based on their beliefs, traits, and preferences. By interviewing 25 mothers, we enhanced our understanding of the necessary elements for an ECD intervention to be acceptable to low-income communities and determined the factors that may affect the usability of the intervention. From the demographic information, the samples in our study were distributed evenly in rural and urban areas and covered a variety of races. The majority of mothers we interviewed reported under ¥5000 (US $750) estimated household income per month. Overall, the samples in our study fitted our targeted ECD intervention, which was mothers of newborn children from the poverty-stricken area.

The results indicated that mothers living in high-poverty regions of China had strong perceived demand for understanding the risk of poor childhood development. They had limited knowledge on ECD and needed evidence-based information. They were concerned that children may experience health problems such as malnutrition and poor cognitive development. From this perspective, we believe that the general guidance on how to deal with common disease and malnutrition as well as anticipatory guidance on development should be easily accessed in the mHealth program to increase the motivation of use. Furthermore, they realized that parent-child interaction played a vital role in ECD. The participants expressed their interests to learn how to read books and play games with their child. Therefore, education information on those aspects should be maintained in the mobile phone–aided intervention.

In terms of likelihood of action, the participants believed that learning parenting knowledge would help them better understand the child’s needs. However, perceived barriers such as having limited time and resources, different opinions in childcare among family members, and distrust of village doctors exist, which may affect whether participants follow the evidence. Therefore, it might help to expand the mobile health program to incorporate training for local doctors and community health workers, in order to build a trustworthy relationship between parents and local health professionals [36]. In addition, many participants lived with their parents, who may influence their decision making, and several participants reported that their husband or mother-in-law made decisions on childcare. Although some indicated that they would prefer to follow the evidence-based information, they were still concerned that their parents or parents-in-law would insist on the traditional beliefs. A potential approach to address this barrier is to create a shared account among family members so that everyone in a household can learn ECD knowledge. It is worth noting that some participants had a high risk of postpartum depression based on the cutoff point of our screening tool, and messages/sections on postpartum mental health support should be incorporated in the ECD intervention.

As for the feasibility and ideas on the ECD app content, we found most participants had access to the internet and smartphones, thus the mobile health program was feasible for this population. However, the participants had relatively low breastfeeding rate/attempts and low health literacy, and thus multimedia (eg, video, graphics, and text) and plain language should be applied to make the content more understandable. Visuals help make the information more persuasive and memorable [37], which is particularly important for people with low health literacy [38]. Plain language with a concise and organized writing style helps participants better understand the messages. Moreover, since the participants indicated that they didn’t have much time due to childcare responsibilities, text message that took more than 5 minutes to read might be lengthy for them. Prior studies have proven that media-assisted interventions, such as video episodes, can increase the reach and reduce the cost and time for parent education; these are also parents’ strongest preferences [39,40]. Therefore, instead of using text, we plan to create 1-minute videos in the new designed app conveying parenting knowledge and early childhood interventions to decrease learning time and make learning more convenient and attractive for the parents.

### Limitations

We did not specifically interview mothers whose children are at older ages, and their needs for parenting education could be different with those moms who have a newborn child. However, we did interview several moms who already have an elder child, so we were able to retrieve some information on this end.

### Conclusion

Our study proposed that mHealth should be considered as one of the techniques in interventions on ECD due to its feasibility, low cost, and potential to cover a wide range of the population. This was the first study to ever understand the beliefs and barriers for a mobile phone–based intervention to improve ECD in a high-poverty region in southwestern China. We conclude that even in poverty-stricken areas, both the technology and the health literacy meet the requirement for the implementation of mHealth programs. Furthermore, this study brought out some potential ideas to make it more feasible when applied on the ground. The results of the study will aid in the development of potentially effective early childhood programs in underserved regions in LMICs.

## Appendix

Multimedia Appendix 1 Interview questions and answers based on the health belief model.

## Abbreviations

ECD: early child development
HBM: health belief model
LMIC: low- and middle-income countries
mHealth: mobile health
