# Supplementary material for: Assessing Feasibility of an Early Childhood Intervention Using Mobile Phones Among Low-Income Mothers of Newborns: Qualitative Interview Study
Source: JMIR Form Res. 2020 May 28;4(5):e17179. doi: 10.2196/17179 (PMC7290447; doi:10.2196/17179)
Supplement: Multimedia Appendix 1 [file formative_v4i5e17179_app1.docx]

Multimedia Appendix 1. Interview Questions and Answers based on the Health Belief Model.

| **Construct** | **Measures** | **Answers** |
| --- | --- | --- |
| Perceived susceptibility | Which of the following children’s development problems are most concerning to you? | - Baby’s health issues (cold, fever, jaundice, sickness) |
|  |  | - Baby’s cognitive development |
|  |  | - Safety issues |
|  |  | - Baby’s behavior problems (worried that the child will be spoiled by grandparents and develops bad habits) |
|  |  | - Breastfeeding and nutrition issues |
|  |  | - Other issues or no response |
|  | Perceived necessity to learn early childhood development knowledge | - Yes |
|  |  | - No |
|  | Which of the following topics in early childhood development are most interesting to you? | - Baby’s health, growth, nutrition, breastfeeding, complementary feeding |
|  |  | - Parent-child interactions; baby’s social-emotional development |
|  |  | - Mental health support for moms |
|  |  | - Baby’s cognitive development |
|  |  | - Baby’s safety issues |
|  |  | - No response |
| Perceived severity | Have you learned parenting knowledge before? What sources? | - Yes. Learned from books and internet |
|  |  | - Yes. Learned from doctors and healthcare professionals |
|  |  | - No. Have not learned parenting knowledge before |
|  | Do you think if you know enough parenting knowledge? | - Having limited parenting knowledge |
|  |  | - Don’t feel the need to learn parenting knowledge |
|  |  | - No response |
|  | Do you have any questions about the parenting knowledge you learned before? | - Thinking parenting information found online is inconsistent |
|  |  | - Valuing suggestions from those whose children were healthier |
|  |  | - Considering parenting information learned from different sources is consistent |
|  |  | - No response |
| Perceived benefits and barriers | Are you expecting to learn parenting knowledge from medical and education professionals? | - Yes. I am willing to learn parenting knowledge from experts |
|  |  | - No. I do not have the need to learn parenting knowledge from experts |
|  | Do you see the benefits of receiving parenting knowledge from professionals? | - Yes, would be beneficial for the child’s development |
|  |  | - No response |
|  | What is your expectation for your child when he/she grow up? | - High expectation for my child: healthy, high achievement in education, good mental well-being, have better life and job than my generation |
|  |  | - Moderate expectation for my child: not as good as urban kids |
|  |  | - Have not thought about the future of my child |
|  |  | - No response |
|  | Do you feel the barriers in following experts’ advice in childcare? | - Do not have barriers in following experts’ advice in parenting |
|  |  | - Having difficulties to following experts’ parenting suggestions due to environmental limitations, time limitations, economic burden and social norms |
|  |  | - No response |
| Maternal  self-efficacy | Who make decisions about childcare in your household? | - Making decisions by myself |
|  |  | - Husband makes the decision about childcare |
|  |  | - Mother-in-law makes the decision regarding childcare |
|  |  | - Making decisions together |
|  | Do you have good relationship with your family members including parents and parents-in-law? | - Having good relationships with family members |
|  |  | - Do not have good relationships with parents-in-law |
|  |  | - No response |
|  | Whose suggestions to follow regarding your child’s health issues (doctors or family members?) | - Follow doctors’ suggestions for health issues |
|  |  | - Follow family members’ suggestions |
|  |  | - Follow both doctors’ and family’s suggestions |
|  |  | - No response |
|  | Are you willing to communicate the knowledge you learned from professionals with your family members, or persuade them in taking the evidence-based advice? | - Yes, willing to communicate with my family on parenting knowledge |
|  |  | - No, because parents-in-law had limited education |
|  |  | - No response |
| Preferences for the design of the intervention | Preferences for the mode of the intervention (smartphone app vs. text messaging) | - Preference for receiving messages via smartphone apps |
|  |  | - No preference for smartphone apps over text messages |
|  | Preference for the frequency of receiving messages | - Every day or twice a week |
|  |  | - Once a week |
|  |  | - Once every other week |
|  | Willingness to read the messages | - Yes |
|  |  | - Yes, will read the messages. Won’t delete the messages |
|  |  | - No response |
|  | Preference for the type of messages (individual tailored messages vs. general messages; willingness to share baby’s personal information) | - Preference for personalized information; willing to share baby’s information (weight, height, date of birth, etc.) |
|  |  | - Preference for general messages; not willing to share baby’s information |
|  |  | - No response |
|  | Willingness to search for answers from professionals when having questions | - Preferring to communicate online or via phone with professionals in large hospitals or in Shanghai remotely |
|  |  | - Preferring to consult with village doctors, local hospitals or local pediatricians in person |
|  |  | - Preferring to look for answers online |
|  | Willingness to share messages with peers or communicate with peers through the app | - Willing to share parenting knowledge with other moms |
|  |  | - Willing to learn from other moms about parenting information |
|  |  | - No response |
| Message contents (participants were shown a sample text message) | Average time to read one message | - Less than 3 minutes |
|  |  | - 3-5 minutes |
|  |  | - More than 5 minutes |
|  | Do you have any suggestions or questions regarding the message? | - Messages are easy to understand |
|  |  | - Adding a platform for interaction and communication |
|  |  | - The recommendations on toys are not accommodate to local environment |
|  |  | - Interested in the parent-child interaction but don’t know how to practice. May add a video in the app |
|  |  | - Not clear about teething symptoms |
|  |  | - Preferring pictures to texts |
|  |  | - Adding reminders for immunization and well-child exams |
|  |  | - Preferring to receive personalized answers |
|  |  | - No comment |
|  | Which one of the four sections are most interesting or useful to you? | - Parent-child activities (games, songs, toy-playing, etc.) |
|  |  | - Child development milestones |
|  |  | - Baby’s weekly life (nurturing, teething, etc.) |
|  |  | - No specific preference (every section is equally useful) |
